# Antidiabetic Effect of Young and Old Ethanolic Leaf Extracts of* Vernonia amygdalina*: A Comparative Study

**DOI:** 10.1155/2016/8252741

**Published:** 2016-05-16

**Authors:** Du-Bois Asante, Emmanuel Effah-Yeboah, Precious Barnes, Heckel Amoabeng Abban, Elvis Ofori Ameyaw, Johnson Nyarko Boampong, Eric Gyamerah Ofori, Joseph Budu Dadzie

**Affiliations:** ^1^Department of Biomedical Sciences, School of Allied Health Sciences, College of Health and Allied Sciences, University of Cape Coast, Cape Coast, Ghana; ^2^Department of Clinical Nutrition and Dietetics, School of Allied Health Sciences, College of Health and Allied Sciences, University of Cape Coast, Cape Coast, Ghana; ^3^Department of Biochemistry, School of Biological Sciences, College of Agriculture and Natural Sciences, University of Cape Coast, Cape Coast, Ghana

## Abstract

The young leaves of* Vernonia amygdalina* are often utilized as vegetable and for medicinal purpose compared to the old leaves. This study was designed to evaluate and compare the antidiabetic effects between ethanolic leaf extracts of old and young* V. amygdalina* on streptozotocin (STZ) induced diabetic rat for four weeks. Preliminary screening of both young and old ethanolic extracts revealed the presence of the same phytochemicals except flavonoids which was only present in the old* V. amygdalina*. Difference in antioxidant power between the young and old leaf extracts was statistically significant (*p* < 0.05). Both leaf extracts produced a significant (*p* < 0.05) antihyperglycaemic effect. Also results from treated rats revealed increasing effect in some haematological parameters. Similarly, the higher dose (300 mg/kg) of both extracts significantly (*p* < 0.05) reduced serum ALT, AST, and ALP levels as compared to the diabetic control rats. Results also showed significant (*p* < 0.05) decrease in LDL-C and VLDL-C in the extract-treated rats with a corresponding increase in HDL-C, as compared to the diabetic control rats. Moreover histopathological analysis revealed ameliorative effect of pathological insults induced by the STZ in the pancreas, liver, and spleen, most significantly the regeneration of the beta cells of the islets of Langerhans in treated rats.

## 1. Introduction

Diabetes is a disorder of carbohydrate, protein, and fat metabolism and can represent an absolute insulin deficiency, impaired release of insulin by the pancreatic *β* cells, inadequate or defective insulin receptors, or production of inactive insulin [1]. Diabetes mellitus can be classified into two main types, type 1 and type 2 [2], with type 1 resulting from the body's failure to produce insulin, and requires one to be injected with insulin [3]. Type 2 diabetes mellitus describes a condition of fasting hyperglycemia that occurs despite the availability of insulin [1]. Medicinal plants are precursors for the synthesis of useful drugs [4] and are used in the management of several disorders including diabetes mellitus [5].

*Vernonia amygdalina* is a shrub common in tropical Africa [6]. It is a member of the Asteraceae family and a source of many local medicines in the West African subregion [7–10]. The young, succulent, and fresh leaves of the plant are mostly preferred in treating ailments such as diabetes, malaria, fever, constipation, and high blood pressure and as laxative, among the indigenous people of the southern part of Ghana. However, due to lack or little knowledge about the correct dose regimen, several complications or side effects may arise during its usage. Various researchers have worked on the phytochemical and antioxidant [11, 12] and the total effect of the entire leaf extract (mixture of both old and young) of the plant in the treatment of various ailments [7, 12–15] but have not compared these parameters between the old and young leaves of this plant.

Thus this study is designed to determine and compare the effect of young and old leaf extracts of* V. amygdalina* in streptozotocin (STZ) induced diabetic rats. To the best of our knowledge, this is the first report that documents the difference in phytochemical, antioxidant, and the overall antidiabetic effect between young and old leaf extracts of this medicinal plant.

## 2. Materials and Method

### 2.1. Study Design

Male and female Sprague Dawley rats (114–143 g) were randomly selected for the experiment. Fifty-six (56) rats were obtained from the Animal House of the University of Cape Coast (UCC).

### 2.2. Study Site

All animal procedures were carried out at the laboratories of the Department of Biomedical Sciences (DBS), UCC.

### 2.3. Maintenance of Animals

The rats were kept in animal rooms at the laboratory where room temperature was maintained at 26 ± 3°C under a 12 hr light-dark cycle. The rats were housed in aluminum cages (12.5 × 16.6 × 7.5 inches) with soft wood shavings as bedding. Animals were fed on food and water* ad libitum*. Test material was administered orally using an oral gavage. Fur dye and picric acid were used to identify the animals. All animal experiments, procedures, and techniques used in this study were conducted in compliance with the National Institute of Health Guidelines for Care and Use of Laboratory Animals. Also, the research work was approved by the Institutional Review Board, University of Cape Coast, Ghana (ethical clearance number: FYP/2015/0040).

### 2.4. Drugs and Chemical

Alanine aminotransferase (ALT), aspartate aminotransferase (AST), alkaline phosphatase (ALP), total bilirubin (TB), total protein (TP), total cholesterol (TC), triglycerides, high density lipoprotein cholesterol (HDL-C), low density lipoprotein cholesterol (LDL-C), and very low density lipoprotein cholesterol (VLDL-C) assay kits (Fortress Diagnostic, UK) were used to assess blood biochemistry. All other chemicals such as glibenclamide (Arzneimittelwerk Godecke, Freiburg, Germany), normal saline, and STZ (Sigma-Aldrich Inc., St. Louis, MO, USA) were of high purity grade.

### 2.5. Preparation of Leaf Extract

*V. amygdalina* leaves were collected from the Botanical Garden of the School of Biological Sciences, University of Cape Coast, and identified by a botanist at the herbarium section, with the voucher specimen # AD 001. The leaves were then grouped into young leaves (YL) and old leaves (OL) using the following criteria; length of YL ranges within 5.10–15.50 mm and width within 1.80–7.20 mm and their corresponding weights range within 0.09–1.20 g. Length of OL ranges within 17.70–30.10 mm and width within 7.20–12.90 mm, with corresponding weights ranging within 1.30–5.30 g. The greenish colouration of the OL was deeper as compared to the YL.

The leaves were separately air-dried for two weeks. They were then pulverized by means of blender and stored in air-tight plastic containers. Five hundred grams (500 g) of the powder of both YL and OL was macerated in separate cold ethanol for 72 hrs. The macerated solution was then decanted. The filtrates were concentrated in vacuum at low temperature of (38–40°C) to about one-tenth the original volume using a rotary evaporator. The concentrates were allowed to be opened in a water bath (40°C) for complete dryness for the ethanol extracts and were then stored in a refrigerator at −4°C. The percentage yields of the extracts were 19% w/w for YL and 22% w/w for OL. Different concentrations (10, 30, and 300 mg/mL) of both extracts were then prepared from the stored extracts for oral administration.

### 2.6. Phytochemical Test

The chemical classes of constituent in the freshly prepared YL and OL extracts were detected using standard phytochemical reagents and procedures as described previously [11, 16].

### 2.7. In Vitro Antioxidant Activity

The radical scavenging capacities of the YL and OL of the plant extracts against 2,2-diphenyl-1-picryl hydrazyl radical (Sigma-Aldrich) were determined by UV spectrophotometry at 517 nm. Radical scavenging activity was measured by method described earlier [11, 17], with slight modification. The concentrations 0.05, 0.1, 0.5, 1.0, 2.0, and 5.0 mg/mL in methanol of the extract in alcohol were prepared. Vitamin C was used as a standard antioxidant at concentrations 0.05, 0.1, 0.2, 0.5, and 0.75 mg/mL. 1 mL of the prepared mixture from each concentration was delivered into a test tube and 3 mL of methanol was added in the tube followed by 0.5 mL of 1 mM DPPH. The same amount of DPPH was used as a blank. The antioxidant property of crude extract of the young* V. amygdalina* was determined against Vitamin C by spectrophotometry at a wavelength of 517 nm. The antioxidant activity was calculated using the following formula: % inhibition = [(*A*_*b*_ − *A*_*a*_)/*A*_*b*_] × 100, where “*A*_*b*_” is absorption of the blank sample and “*A*_*a*_” is the absorption of the extract.

### 2.8. Induction of Diabetes

After one week of acclimatization period, DM was induced experimentally in rats by multiple intraperitoneal injections of 40 mg/kg STZ dissolved in citrate buffer daily for three days. Control rats were intraperitoneally injected with the citrate buffer alone. After the three days of STZ treatment, blood samples were taken from tail vein using a tail clip and glucose levels measured with a glucometer (Accu-CHEK). Rats with nonfasting glucose levels higher than 15 mmol/dL were considered as being diabetic and were included in the study.

### 2.9. Experimental Design

A total of 56 experimental rats were used to assess the effect of the leaf extracts on the experimental rats: 49 STZ induced diabetic rats plus 7 normal control rats. Animals were divided into eight (8) major groups and housed under controlled environmental conditions. Rats were divided into the following groups:

 Group 1 (normal control): non-STZ induced rats.
 Group 2 (negative control), STZ induced diabetic rats: normal saline.
 Group 3 (positive control), STZ induced diabetic rats: standard drug (glibenclamide 10 mg/kg).
 Group 4, STZ induced diabetic rats: extract of old* V. amygdalina* (10 mg/kg).
 Group 5, STZ induced diabetic rats: extract of old* V. amygdalina* (30 mg/kg).
 Group 6, STZ induced diabetic rats: extract of old* V. amygdalina* (300 mg/kg).
 Group 7, STZ induced diabetic rats: extract of young* V. amygdalina* (10 mg/kg).
 Group 8, STZ induced diabetic rats: extract of young* V. amygdalina* (30 mg/kg).
 Group 9, STZ induced diabetic rats: extract of young* V*.* amygdalina* (300 mg/kg).

### 2.10. Assessment of Fasting Plasma Glucose Levels and Body Weight Measurement

The body weight measurement and fasting plasma glucose levels were assessed before and during administration of the extracts weekly, till the end of the study.

### 2.11. Haematological and Biochemical Parameters

Experimental rats were sacrificed and blood was dispensed into EDTA tubes and then analyzed for haematological indices such as red blood cells (RBCs), haemoglobin (Hb), haematocrit (HCT), mean corpuscular volume (MCV), mean corpuscular haemoglobin (MCH), mean corpuscular haemoglobin concentration (MCHC), monocytes (MON), neutrophils (NEU), and lymphocytes (LYM) using an automated haematology analyzer (MSLAB 12, China). Another portion of blood was collected in empty vacutainer tubes and centrifuged at 13000 rpm for 5 mins. The serum was then retrieved and biochemical parameters such as AST, ALT, ALP, triglycerides, TB, TP, TC, triglycerides, HDL-C, LDL-C, and VLDL-C in serum were analyzed using commercially available kits and a standard BS-120 Mindray Chemistry Analyzer.

### 2.12. Histological Analysis

The rats were dissected and the liver, spleen, and pancreas were removed and observed for evidence of gross pathology. For light microscopic examination, tissue samples were immediately fixed in 10% phosphate buffered formalin, processed using an automated tissue processor (Leica TP 1020, Germany) and finally embedded in paraffin wax. The tissue blocks were then cut into serial sections with thickness of 6 *μ*m using the rotary microtome. The sections were deparaffinized and subsequently stained with haematoxylin and eosin (H&E) as described earlier [18]. The pancreatic, hepatic, and splenic microscopic architecture of experimental rats on the H&E stained slides were histopathologically examined.

### 2.13. Statistical Analysis

GraphPad Prism Version 5.0 for Windows (GraphPad Software, San Diego, CA, USA) was used for data analysis. Data were presented as mean ± SEM on line graphs and tables. For comparing control and treatment group, independent samples *t*-tests for significance of differences were used. To compare biological effects of the treatment, analysis of variance (ANOVA) was used. Post hoc analysis was also used to identify the source of the variation. *p* values less than 0.05 were considered statistically significant.

## 3. Results

### 3.1. Phytochemical Screening

Preliminary screening of ethanolic extracts of both young and old* V. amygdalina* leaves revealed the presence of alkaloids, tannins, saponins, cardiac glycosides, terpenes, and steroids (Table 1). However flavonoids were absent in the YL but present in the OL of the plant extract.

### 3.2. Antioxidant Activity

The crude extract of YL and OL of* V. amygdalina* exhibited a concentration-dependent antioxidant activity in the presence of nitric oxide (NO) (Table 2). At high concentration (1.0 mg/mL), the antioxidant activity of ascorbic acid (Vitamin C) was found to be 1.27 and 2.15 times greater than the YL and OL extract, respectively, with YL 1.69 times greater than OL. These ratios between the standard and the extracts increased drastically (3.04 and 4.29 for YL and OL, resp.) when the concentration was reduced to 0.05 mg/mL, once more with OL 1.41 times less potent than YL. Though statistically significant (*p* < 0.05), the ratios showed that the extracts have high free radical scavenging properties at higher concentrations, with YL having a higher degree of radical scavenging activity than OL.

### 3.3. Effect of Old and Young Ethanolic Leaf Extract of* V. amygdalina* on Weight of Diabetic Rats

Rats in the negative control group all manifested significant loss in body weight. In the normal control group and treated rats, though not statistically significant (*p* > 0.05), there was appreciable gain of weight (Table 3) except in rats that received 300 mg/kg YL extract. This indicates that action of* V. amygdalina* on body weight may be dose dependent.

### 3.4. Effect of Old and Young Ethanolic Leaf Extract of* V. amygdalina* on Haematological Parameters

The effects of STZ induced diabetes on haematological parameters were investigated and compared with similar parameters of normal nondiabetic rats as shown in Table 4. Following induction of diabetes in the rats, most of the haematological parameters were significantly reduced in diabetic rats than in the nondiabetic control rats. It also shows the haematological parameters of diabetic rats following treatment with 10 mg/kg, 30 mg/kg, and 300 mg/kg of YL and OL extracts of* V. amygdalina*.

Comparison between the respective doses of YL and OL leaf extracts with respect to the normal revealed similar effect. RBCs, Hb level, and neutrophils decreased significantly in the diabetic control group as well as the treated groups compared to the normal control. There was no significant difference in the haematological parameters in any of the three groups of both young and old* V. amygdalina,* respectively.

### 3.5. Effect of Old and Young Ethanolic Leaf Extract of* V. amygdalina* on Biochemical Parameters

All doses of YL and OL, including the positive control, significantly (*p* < 0.05) reduced the elevated levels of the liver enzymes (AST, ALT, and ALP) compared to the negative control. Though not significant for the other liver function tests (TP and TB), decreased TP was elevated while elevated levels of TB were appreciably reduced in the treated groups as compared to the diabetic control group. Similarly, the diabetic control group showed elevated levels of TC, triglycerides, LDL-C, and VDL-C with lowered levels of HDL-C (Table 5). Serum levels of LDL-C and VDL-C were significantly reduced (*p* < 0.05) with a corresponding increase in HDL-C in treated rats. Comparing the different doses of the two groups (YL and OL), though not significant, the normalization of the biochemical indices was higher in the YL (10–300 mg/kg) than in the OL (10–300 mg/kg) as compared to the normal control group, except for ALP, where the high dose of OL (300 mg/kg) showed a better curative effect (532.21 ± 2.11 U/L) than the 300 mg/kg YL (528.25 ± 6.59 U/L).

### 3.6. Effect of Young and Old Leaf Extract of* V. amygdalina* on Hyperglycemia

**Figure 1:**
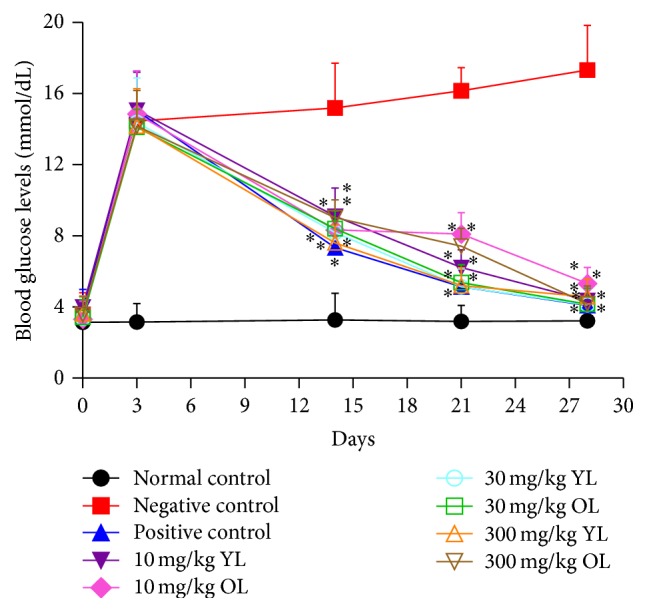
Blood glucose levels before and after administration of both old and young leaf extracts of* V. amygdalina.* Values are expressed as mean ± SEM (*n* = 7). Note: *p* < 0.05 was considered statistically significant. (*∗*) = statistical difference between treated groups and negative control.

A general increase in the blood glucose level was observed in the rat following the administration of STZ, with mean glucose levels ranging within 15.0–15.60 mmol/dL. Treatment of the hyperglycemic rats with the different doses (10, 30, and 300 mg/kg) of both YL and OL produced a significant (*p* < 0.05) antihyperglycaemic effect on the 14th, 21st, and 28th day of the experiment compared to the negative control group as shown in Figure 1. The highest reduction (4.10 ± 1.60 mmol/dL) was observed in the positive control group (treated with glibenclamide) and the lowest reduction (5.33 ± 0.90 mmol/dL) was observed in the 10 mg/kg OL of the plant extract on the 28th day of the experiment. However the diabetic control group manifested continuous increase in glucose levels from day 0 to the 28th day (Figure 1).

### 3.7. Histopathological Evaluation

**Figure 2:**
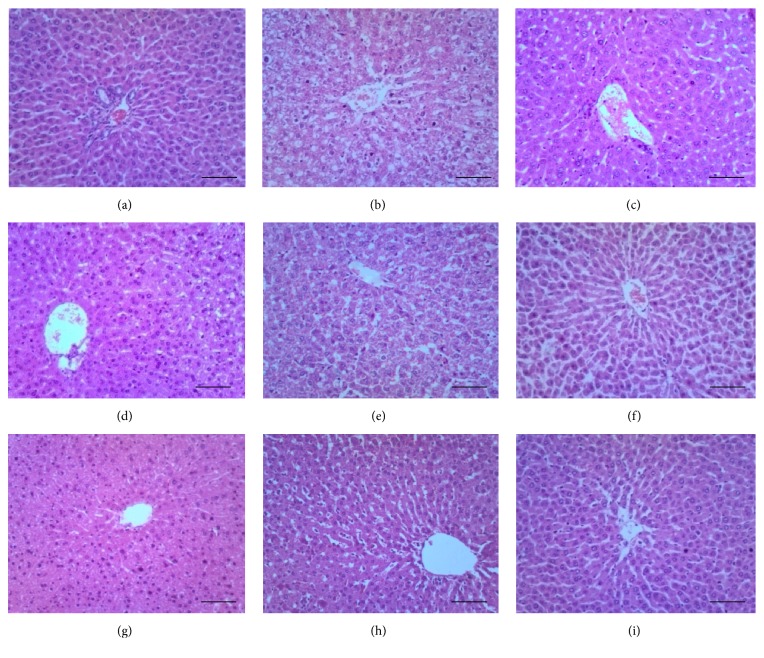
Photomicrograph of liver sections. (a) Normal control: normal polygonal shape of hepatocytes, devoid of cellular injuries. (b) Negative control: it shows fatty change (cytoplasmic halo), slightly edematous tissue section, activated Kupffer cells, and basophilic and pyknotic nuclei of some hepatocytes surrounding the central vein (CV). (c) Positive control: normal hepatocytes with increased eosinophilia, accompanied with mild leucocytosis. (d) 10 mg/kg YL: normal hepatocytes with focal areas of necrosis. (e) 10 mg/kg OL: reduced fatty change as compared to the negative control, with mild oedema. (f) 30 mg/kg YL: section depicts normal cells of the liver, with reduced eosinophilia of cytoplasm of hepatocytes. (g) 30 mg/kg OL extract: focal areas of necrosis with reduced fatty change. (h) 300 mg/kg YL: focal areas of necrosis with mark sinusoidal leucocytosis. (i) 300 mg/kg OL extract: normal hepatocytes with very mild leucocytosis (H&E stain 100x). Bar = 20 *μ*m.

Histopathological examination of the liver from the normal control group (Figure 2(a)) revealed normal histological structure of hepatocytes, hepatocytes polygonal in shape, tightly parked, containing basophilic central rounded nuclei separated by the hepatic sinusoids, and with the presence of nonactivated Kupffer cells within the walls of the sinusoids. Section from the negative control group (Figure 2(b)) shows fatty changes (cytoplasmic vacuolations) as a result of fat accumulation within cytoplasm of hepatocytes, accompanied by activated Kupffer cells (resident reticuloendothelial cells) and basophilic nuclei of some hepatocytes. Fatty changes manifested as abnormal swelling of hepatocytes together with microvesicular and macrovesicular changes within cytoplasm and with some of these vesicles merging together extracellularly to form fatty cysts (extrahepatic cysts). Hepatocytes also manifested different stages (pyknosis, karyorrhexis, and karyolysis) of necrosis. The positive control (Figure 2(c)) had normal hepatocytes with increased eosinophilia of the cytoplasm, accompanied with mild leucocytosis within the hepatic sinusoids. Section from Figure 2(d) (10 mg/kg YL) shows normal hepatocytes with focal areas of necrosis, while Figure 2(e) (10 mg/kg OL) on the other hand had reduced fatty changes within the cytoplasm of the hepatocytes as compared to the negative control, accompanied by mild oedema seen as dilation of sinusoids.

In Figure 2(f) (30 mg/kg YL), section depicts normal cells of the liver, with slightly reduced eosinophilia of their cytoplasm, and this gives clear evidence of cellular regeneration as compared to the negative control group.  Figure 2(g) (30 mg/kg OL) shows focal areas of necrosis with reduced fatty change (mild form of microcytoplasmic vacuolations present) and this suggests cellular regeneration, but being less pronounced as compared to the 30 mg/kg YL. Similarly, Figure 2(h) (300 mg/kg YL) shows focal areas of necrosis with mark sinusoidal leucocytosis accompanied by mild form of oedema and activated Kupffer cells. For Figure 2(i) (300 mg/kg OL), there was evidence of total regeneration, with section entirely populated by normal hepatocytes as compared to the normal control group, though there were very mild leucocytoses. Difference between Figures 2(h) and 2(i) was subtle, only that the former had increased sinusoidal leucocytosis accompanied by a very mild oedema.

**Figure 3:**
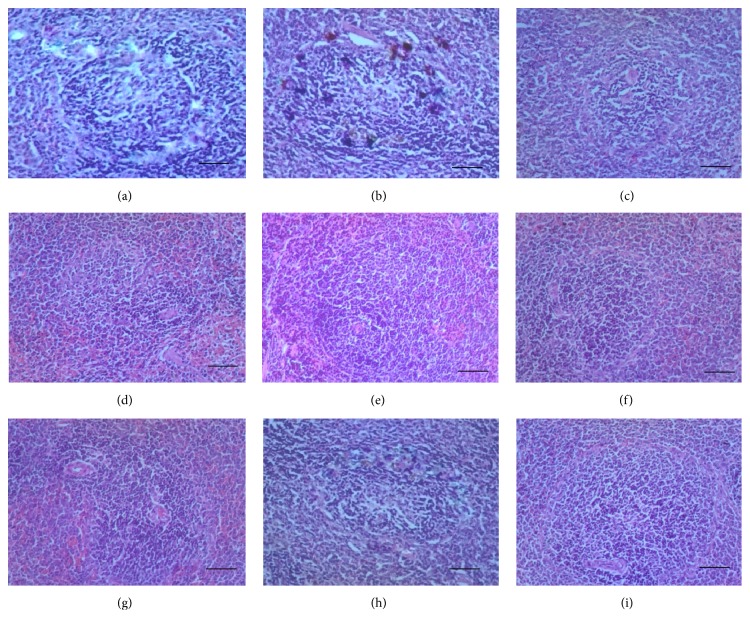
Photomicrograph of spleen sections. (a) Normal control: normal architecture of spleen with presence of marginal zone between red and white pulp. (b) Negative control: hypercellularity and haemosiderosis with loss of marginal zone and dilatation of blood vessels. (c) Positive control: it shows no sign of hypercellularity or haemosiderosis. Sections (d) (10 mg/kg YL), (e) (10 mg/kg OL), (f) (30 mg/kg YL), (g) (30 mg/kg OL), (h) (300 mg/kg YL), and (i) (300 mg/kg OL) all show evidence of hypercellularity of resident cells and absence of haemosiderosis (H&E stain 100x). Bar = 20 *μ*m.

Examined spleen section from the normal control group (Figure 3(a)) shows normal architecture of spleen with presence of marginal zone between red and white pulp, devoid of haemosiderosis. On the other hand, section from the negative control (Figure 3(b)) shows hypercellularity and haemosiderosis with loss of marginal zone (between red and white pulp) and dilatation of blood vessels. The haemosiderosis depicts increased destruction of RBCs with the hypercellularity conferring evidence of hyperplasia of the lymphoreticular cells due to hyperactivity against the STZ.

The extract-treated groups (10–300 mg/kg of both YL and OL) showed hypercellularity of resident cells but no haemosiderosis (Figures 3(d)–3(i)). The positive control, Figure 3(c) (treated with standard drug), however, showed no sign of hypercellularity or haemosiderosis.

**Figure 4:**
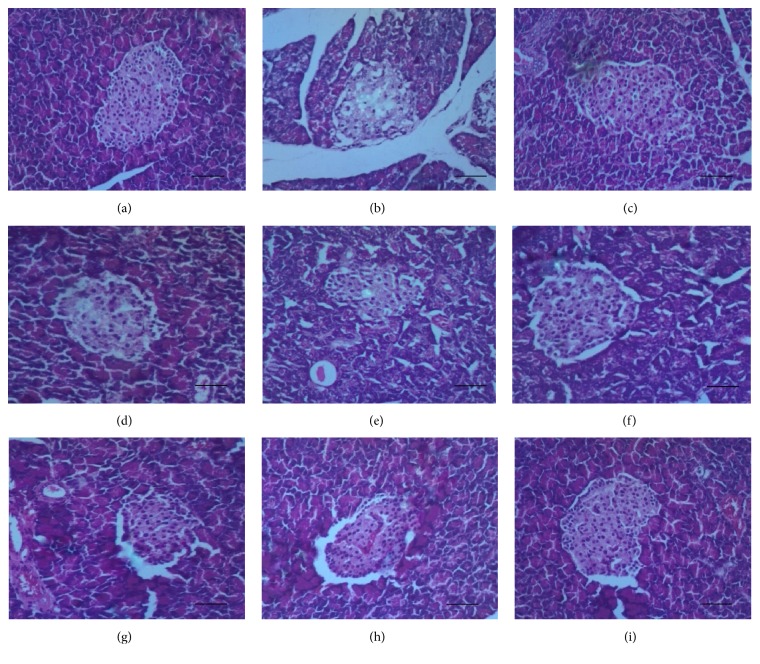
Photomicrograph of histological sections of pancreas. (a) Normal control: section shows normal histology of pancreas with high cellularity of the islet of Langerhans. (b) Negative control: islets show highly reduced cellularity of the endocrine islets with inter- and intracytoplasmic vacuolations. (c) Positive control: it has normal arrangement of islet cells and the cellularity is likened to that of the normal control. (d) 10 mg/kg YL: reduction of cells in the middle portion of the islets of Langerhans. Sign of little regeneration. (e) 10 mg/kg OL: it also shows atrophy of islet with little regeneration of resident cells. (f) 30 mg/kg YL: islet has increased number of cells as compared to the normal control but being less compact. (g) 30 mg/kg OL: it also shows sign of regeneration but cellularity is less pronounced as compared to the 30 mg/kg YL. (h) 300 mg/kg YL: it shows sign of total regeneration of cells of the islet. (i) 300 mg/kg OL: it displays sign of total regeneration of the islets of Langerhans (H&E stain 100x). Bar = 20 *μ*m.

Analyzed histological section of the pancreas from the normal control group (Figure 4(a)) shows normal histological architecture of pancreas with high cellularity of the islet of Langerhans (high cellularity of the islet which obviously denotes high number of beta (*β*) cells and non-*β*-cells). There is also compact arrangement of cells of the islets. Section from the diabetic control experimental rats (Figure 4(b)) shows highly reduced cellularity and cytoplasmic degranulation, accompanied by hydropic swelling and vacuolations, with the few cells present, sparsely arranged. The loss of cells mostly from the middle portion of the endocrine islets of Langerhans thus displays localized degeneration of cells at the center where *β*-cells of the islets are mostly concentrated. Thus the *β*-cells that are responsible for the secretion of insulin were highly reduced, giving evidence of *β*-cells insufficiency. Positive control rats treated with glibenclamide (Figure 4(c)) also manifested complete restoration of the islet of Langerhans. Has normal arrangement of islet cells and the cellularity is likened to that of the normal control. Histological section from rats treated with 10 mg/kg YL (Figure 4(d)) shows reduction of cells in the middle portion of the islets of Langerhans which depicts evidence of little regeneration, with regularly arranged peripheral islet cells, similar to animals treated with 10 mg/kg OL (Figure 4(e)), with the later showing signs of atrophy of islets. Moreover with rats treated with 30 mg/kg YL (Figure 4(f)), islet has increased number of cells as compared to the normal control but being less compact.  Figure 4(g) (30 mg/kg OL) reveals sign of regeneration but cellularity is less pronounced as compared to the 30 mg/kg.  Figure 4(h) (300 mg/kg YL) shows sign of regeneration of cells of the islet, with section from Figure 4(i) (300 mg/kg OL) manifesting sign of complete regeneration of the islet cells. 30 mg/kg YL, 300 mg/kg YL, and 300 mg/kg OL together with the positive control group displayed high regenerational abilities of the islets.

It was observed that the regenerational abilities of the extracts were variable, in relation to the direct degree of damage and residual effects of the damage on hepatocytes of the liver, the islets of the pancreas, and lymphoreticular cells of the spleen.

## 4. Discussion

Phytochemical constituents available in the ethanolic extracts of both YL and OL of* V. amygdalina* leaf in this study revealed the presence of alkaloid, tannins, saponins, cardiac glycosides, terpenoids, glycosides, and reducing sugars, which correlates with research done previously [7], using fresh leaf extracts of the plant. An intriguing finding in this study was the presence of flavonoids, present in the OL extract but absent in the YL extract. The former statement (presence of flavonoids) conforms to reports from other researchers [11, 12] who detected flavonoids in* V. amygdalina* and did not specify whether they were old or young leaves of* V. amygdalina*. This difference can be attributed to the fact that some of the phytochemicals present in the leaf may be synthesized during the later stages, as the leaves mature.

Terpenoids reduce blood glucose through insulin-like activity and inhibition of gluconeogenesis and glycogenolysis [19]. Saponin from* Entada phaseoloides* was reported [20] to alleviate hyperglycemia associated oxidative stress in type 2 diabetes. Research also shows that tannins extracted from different plant materials exhibited *α*-amylase and *α*-glucosidase inhibitory activities [21], thus decreasing glucose transport through the intestinal epithelium. Similarly flavonoids [22] and alkaloids [23] have been reported to inhibit *α*-glucosidase, hence their remarkable additive effect in reducing blood glucose level in the current study.

Results also show that the extracts have high free radical scavenging property at high concentrations, with YL having a higher degree of radical scavenging activity than OL which is a possible suggestion of its potent antiglycaemic and antilipidaemic effect. Enormous increase in quantity of one or more bioactive compounds (phytochemicals) in the YL may probably exhibit the corresponding increase in the antioxidant power realized, and this is demonstrated in the high DPPH scavenging activity of the young. The cumulative effect of this may be seen in the efficient scavenging of free radicals produced in the animals; thus this compound effectively augments the endogenous antioxidant defense mechanisms as reported elsewhere [24], producing a net reparative effect higher than the OL in the treated rats, efficient enough in treating free radical induced pathological insults.

The positive control group and the extract-treated rats all manifested a significant reduction in blood glucose levels as compared to the negative control. The highest reduction (4.10 ± 2.60 mmol/dL) was observed in the positive control group (treated with glibenclamide), followed by treated rats that received 30 mg/kg YL (4.13 ± 0.46 mmol/dL) of the plant extract. However, the diabetic control group manifested continuous increase in glucose levels from day 0 to the 28th day. The result of this study confirms earlier report by other researchers [7, 12] on the hypoglycemic effect of the leaf extract of* V. amygdalina* in rat. STZ is a diabetogenic agent which induces diabetes by damaging the pancreatic *β*-cells of the islets leading to hyperglycemia [25]. This study correlates with previous research findings [26, 27], where antidiabetic medicinal plants significantly reduced the high blood glucose level in STZ induced diabetic rats.

Furthermore, the study revealed a decrease in a number of haematological parameters especially RBCs, neutrophil, and monocyte count in the negative control group, confirming work done by Ajagbonna et al. [28], who reported that ingestion of medicinal compounds or drugs can alter the normal range of haematological parameters and might be due to inhibited haematopoiesis or an increase in the destruction of RBCs [29] by the action of the STZ. Though changes in the other parameters (Hb, HCT, MCV, MCH, MCHC, and lymphocytes) were subtle, treatment of the diabetic rats with respective doses of 10, 30, and 300 mg/kg of both YL and OL extract of* V. amygdalina* together with 10 mg/kg glibenclamide brought parameters to near normal as compared to the normal control group. This confirms reports by researchers elsewhere [12, 30]. Most of the haematological parameters, both old and young extract* V. amygdalina,* shared similar effect, but with the 30 mg/kg YL, demonstrating high ameliorative effect, compared to the other doses. The high dose (300 mg/kg) of the YL showed the least in normalization of the haematological indices. This adverse effect may probably be as a result of the high antioxidant capacity in this dose and is in line with reports elsewhere [24, 31], where increase in antioxidant levels above its physiological dose had cytotoxic effect, also consistent with earlier finding [13] which suggested that methanolic leaf extract of* V. amygdalina* had the potential of adversely affecting haematological indices. The observed significant difference between the negative control and treated rats in some of the enzyme level associated with liver function test (ALT, AST, and ALP) as well as HDL-C, LDL-C, and VLDL-C levels, and this is in agreement with reports from previous research works [26, 32] where STZ was used as the diabetogenic agent. The increase levels in AST, ALT, and ALP in blood serum in this study suggest hepatocellular damage as a result of STZ toxicity. It may also suggest that posthepatic damage such as biliary stone formation and biliary tract damage may compound this abnormal rise in lipid profiles and liver enzyme levels in the blood. However, treatment with the different doses of YL and OL of* V. amygdalina* and glibenclamide significantly reduced the ALT, ALP, and AST levels in blood of the STZ induced diabetic rats, indicating the ameliorative effect of the extract on the hepatocytes of the liver after action of the injurious agent (STZ) in the diabetic state, which is in agreement with research work carried elsewhere [12, 27]. The reduced HDL-C levels in the negative control group is a result of insufficiency in fatty acid metabolism, increased gluconeogenesis, and high production of ketone bodies in the diabetic state, which may consequently give rise to hypercholesterolemia and hypertriglyceridemia which are the most commonly observed lipid abnormalities in diabetes [33].

The observed weight gain, though the differences were not statistically significant, was exhibited by the ethanol extract of both YL and OL of* V. amygdalina* leaves with their respective doses (10–300 mg/kg) together with the positive control.

Hence, the ability of the extracts to significantly reduce the high blood glucose levels and effectively increase the body weight of the diabetic rats may be attributed to its antihyperglycaemic effect. Other recent scientific findings [12, 27] attest to this result, with evidence of weight gain after treatment with medicinal plant preparations. The negative control on the other hand showed weight loss. The decrease in body weight associated with diabetes mellitus has been attributed to the gluconeogenesis giving rise to increased muscle wasting and loss of proteins in tissues [34], and this could account for the loss of weight seen in the negative control group.

Histopathological results showed normal pancreatic acini and islets of Langerhans in the normal control group, with the negative control group showing evidence of nuclei degeneration and cytoplasmic changes in the cells of the islets, specifically within the middle portion of the islets where there was reduced cellularity. This concurs with previous reports where STZ induced diabetic rats showed atrophy and focal necrosis within islet cells of Langerhans [26, 27].

Both extracts (young and old) showed regeneration of the *β*-cells of the pancreas with respect to the doses, and this fact is supported by the decrease in blood glucose levels. These changes were consistently observed in all animals in these groups. However 30 mg/kg YL and 300 mg/kg OL of the extract manifested the greatest pancreas regenerative ability compared to the other groups. This study affirms the work done by Ojiako and Nwanjo [30], who said that the methanol leaf extract* V. amygdalina* has protective effect as evidenced by minimal necrotic changes seen in injured tissues after extract administration.

The increase in lipid profile parameters (TC, triglycerides, LDL-C, and VDL-C) and liver enzyme parameters (AST, ALT, and ALP) in the diabetic control rats in our results could be explained by the fatty changes (cytoplasmic vacuolation) and hepatocytic degeneration observed in the liver sections. This may be as a result of the STZ conversion to metabolites in the liver that caused catalytic membrane phospholipid peroxidation, breaking down the endoplasmic reticulum, which ultimately caused reduced lipid export from the liver cells and thus accumulation of lipid in the hepatocytes, as described elsewhere [35, 36]. This is in agreement with other reports elsewhere, where liver sections of STZ induced diabetic rats showed fatty changes [26, 37] evidenced as cytoplasmic vacuolizations [38]. Similarly, other liver pathologies such as hepatocellular necrosis and infiltration of nonspecific inflammatory cells which concurs with the liver tissue damage from the negative control group realized in our work were also reported in previous works [26, 27, 38]. However, the different doses of the YL and OL extracts histopathologically showed evidence of hepatoprotective abilities, with the 30 mg/kg YL and 300 mg/kg of both YL and OL, showing the highest curative effect in the hepatocytes of the liver. This finding is comparable to research works elsewhere [16, 26, 30, 39], where medicinal plant extracts demonstrated the ability to enhance repair of hepatocellular damage, with each researcher inducing the cellular damage with different injurious agent.

Haemosiderosis observed in spleen sections of the negative control group may be as a result of the toxic effect of the STZ on the RBCs as mentioned earlier. This pathological effect manifested as an accumulation of iron pigments, depicting increased destruction of RBCs [35], which can be linked to the low levels of this blood cell type in this group from results obtained. In addition, there was hypercellularity in spleen section of both negative and extract-treated groups causing diminished marginal zone between the red and white pulp. This overproliferation may be a result of immune reactions elicited by the resident lymphoreticular cells [40] against the STZ and the extracts. Clearance of haemosiderin was dose dependent, with tissue sections from the medium dose (30 mg/kg) of YL and the higher doses (300 mg/kg of both YL and OL) demonstrating total absence of the defect. This shows the ability of the extract to reduce the effect of toxicity induced by aetiological agents at the tissue level.

Gross anatomical inspection showed hypertrophy of spleen of rats that received 300 mg/kg of both YL and OL of the extracts. Research work from other authors [8, 16] using different plant extracts in experimental animal models gives credence to this finding. Perhaps it could be linked to increased hyperplasia (hypercellularity) of reticuloendothelial cells as explained earlier in this work.

In summary, for the lower concentrations, 10 and 30 mg/kg YL of the extracts were more efficacious than the OL (10 and 30 mg/kg OL) as compared to the normal control group for blood and tissue parameters that were analyzed. For the high concentrations, 300 mg/kg OL exhibited a better curative ability for haematological parameters than the other doses, but with reduced ameliorative effect for the biochemical parameters as compared to 300 mg/kg YL. The observations taken together add credence to the assertion that leaf extracts of* V. amygdalina* have the ability to protect against STZ induced tissue degeneration in diabetes and clearly justifies the use of the younger leaves as potent antidiabetic agents.

## 5. Conclusion

From the results obtained in this study, it can be concluded that both YL and OL extract of* V. amygdalina* possess antidiabetic properties which may explain the traditional use of this plant for management of diabetes mellitus and its complications but differ in their phytochemical compositions, antioxidant capacity, and pancreatic and hepatic regenerational abilities.

## Figures and Tables

**Table 1:** Preliminary screening of ethanolic leaf extract of *V. amygdalina*.

Phytochemicals	OL	YL
Flavonoids	+++	−−−
Alkaloids	+++	+++
Saponins	+++	+++
Tannins	+++	+++
Glycosides	+++	+++
Reducing sugars	+++	+++
Terpenoids	+++	+++
Anthraquinones	−−−	−−−

+++ = detected; −−− = not detected; OL = old leaf; YL = young leaf.

**Table 2:** Total antioxidant activity between the old and young leaf extract.

Concentration	Vitamin C (mean ± SEM)	YL (mean ± SEM)	OL (mean ± SEM)
1.0 mg/mL	94.43 ± 0.62	74.21 ± 3.19^*∗*^	44.01 ± 0.69^#a^
0.5 mg/mL	89.33 ± 0.74	67.05 ± 2.15^*∗*^	32.95 ± 1.66^#a^
0.1 mg/mL	71.76 ± 7.82	29.11 ± 3.19^*∗*^	21.94 ± 0.21^#a^
0.05 mg/mL	64.32 ± 1.72	21.15 ± 1.05^*∗*^	14.99 ± 0.86^#a^

(*n* = 7) *p* < 0.05 was considered statistically significant. *∗* = statistical difference between control and YL; # = statistical difference between control and OL; a = statistical difference between YL and OL.

**Table 3:** Effect of old and young ethanolic leaf extract of *V. amygdalina* on body weight of experimental rats.

Experimental groups	Initial body weight (g)	Final body weight (g)
Normal	130.91 ± 16.0	139.99 ± 14.30
Negative control	143.58 ± 11.50	117.03 ± 18.03
Positive control	141.20 ± 19.30	169.94 ± 20.12
10 mg/kg YL	135.21 ± 21.03	139.13 ± 43.20
30 mg/kg YL	123.16 ± 12.06	154.34 ± 26.71
300 mg/kg YL	114.22 ± 22.97	119.22 ± 20.16
10 mg/kg OL	116.90 ± 16.12	128.95 ± 22.40
30 mg/kg OL	125.07 ± 14.04	137.23 ± 17.34
300 mg/kg OL	132.66 ± 62.50	146.21 ± 9.32

Values are expressed as mean ± SEM (*n* = 7).

**Table 4:** Effect of young and old ethanolic leaf extracts of *V. amygdalina* on haematological parameters.

Parameters	Normal control	Negative control	Positive control	10 mg/kg YL	30 mg/kg YL	300 mg/kg YL	10 mg/kg OL	30 mg/kg OL	300 mg/kg OL
RBC (M/*μ*)	8.20 ± 0.18	6.50 ± 0.03	8.0 ± 0.64	7.76 ± 0.30	8.61 ± 0.16	7.20 ± 0.12	8.26 ± 0.16	7.38 ± 1.12	7.19 ± 4.02
Hb (g/dL)	16.22 ± 0.25	13.04 ± 1.05	14.7 ± 1.03	14.05 ± 0.97	15.20 ± 0.30	13.53 ± 0.14	14.11 ± 0.19	13.7 ± 2.11	14.26 ± 0.21
HCT (%)	48.40 ± 0.23	37.2 ± 2.01	42.8 ± 3.54	43.73 ± 0.14	49.01 ± 1.10	38.7 ± 0.38	45.4 ± 0.86	39.3 ± 0.45	391.7 ± 0.24
MCV (fL)	53.04 ± 0.62	53.01 ± 3.02	53 ± 2.07	55.01 ± 0.40	53.11 ± 0.13	54.03 ± 0.12	55.12 ± 0.45	53.4 ± 2.42	56.29 ± 0.10
MCH (pg)	17.82 ± 0.11	18.6 ± 0.12	18.4 ± 0.54	16.50 ± 0.13	18.11 ± 0.30	18.30 ± 0.14	14.08 ± 0.26	17.2 ± 0.22	18.06 ± 0.47
MCHC (g/dL)	33.40 ± 0.43	35.9 ± 1.21	34.4 ± 0.34	29.20 ± 0.24	34.09 ± 0.21	35.40 ± 3.01	29.90 ± 0.51	32.3 ± 0.21	37.22 ± 0.13
LYM (%)	55.90 ± 0.09	52.5 ± 0.32	46.9 ± 4.03	51.14 ± 6.12	48.26 ± 0.36	47.1 ± 0.33	52.40 ± 0.11	44.9 ± 0.05	57.12 ± 0.09
MON (%)	1.46 ± 0.14	14.21 ± 0.18	1.60 ± 0.03^*∗*^	2.60 ± 0.11^*∗*^	2.12 ± 0.53^*∗*^	8.20 ± 2.13^*∗*^	5.10 ± 0.16^*∗*^	4.21 ± 2.03^*∗*^	6.10 ± 2.31^*∗*^
NEU (%)	39.10 ± 3.03	30.20 ± 1.12	40.1 ± 0.25^*∗*^	33.12 ± 2.10	40.80 ± 0.17^*∗*^	33.40 ± 0.05	31.30 ± 0.33	35.12 ± 0.41	37.31 ± 5.10^*∗*^

Values are expressed as mean ± SEM (*n* = 7); *p* < 0.05 was considered statistically significant. *∗* = statistical difference between treated groups and negative control. Red blood cells, RBC; haemoglobin, Hb; haematocrit, HCT; mean corpuscular volume, MCV; mean corpuscular haemoglobin, MCH; mean corpuscular haemoglobin concentration, MCHC; monocytes, MON; neutrophils, NEU; and lymphocytes, LYM.

**Table 5:** Serum biochemistry parameters in rats orally treated with YL and OL extracts of *V. amygdalina*.

Parameters	Normal control	Negative control	Positive control	10 mg/kg YL	30 mg/kg YL	300 mg/kg YL	10 mg/kg OL	30 mg/kg OL	300 mg/kg OL
*Liver function test*									
AST (U/L)	156.48 ± 6.06	321 ± 3.04	165.02 ± 2.23^*∗*^	243.39 ± 4.17^*∗*^	201.92 ± 5.10^*∗*^	166.04 ± 7.18^*∗*^	248.03 ± 2.01^*∗*^	208.05 ± 3.30^*∗*^	189.20 ± 6.10^*∗*^
ALT (U/L)	49.00 ± 2.56	192 ± 5.01	69.20 ± 1.25^*∗*^	112.08 ± 3.30^*∗*^	98.50 ± 1.86^*∗*^	75.13 ± 3.07^*∗*^	101.32 ± 4.53^*∗*^	87.25 ± 0.17^*∗*^	69.17 ± 12.6^*∗*^
ALP (U/L)	503.88 ± 2.79	603 ± 3.43	538.20 ± 0.32^*∗*^	573.25 ± 1.07^*∗*^	561.75 ± 4.38^*∗*^	528.25 ± 6.59^*∗*^	580.03 ± 1.32^*∗*^	566.11 ± 0.59^*∗*^	532.21 ± 2.11^*∗*^
TP (g/dL)	88.16 ± 0.20	77.02 ± 41.01	83.06 ± 2.56	80.30 ± 3.12	81.07 ± 3.30	86.03 ± 1.84	80.22 ± 0.25	80.29 ± 1.04	84.23 ± 6.52
TB (Umol/L)	15.01 ± 0.44	16.25 ± 0.73	15.41 ± 0.22	16.12 ± 0.45	16.01 ± 1.45	15.82 ± 0.06	16.15 ± 1.12	16.08 ± 0.13	15.90 ± 1.54

*Lipid profile*									
TC (mmol/L)	4.02 ± 0.26	8.04 ± 3.04	4.54 ± 3.02	6.28 ± 0.39	5.31 ± 0.20	4.26 ± 0.32	7.01 ± 0.23	5.81 ± 1.62	4.91 ± 2.01
Triglyceride (mmol/L)	1.48 ± 0.07	2.51 ± 0.04	1.82 ± 0.06	2.23 ± 0.13	1.85 ± 0.14	1.70 ± 0.09	2.40 ± 1.05	1.87 ± 0.05	1.08 ± 0.11
HDL-C (mg/dL)	51.30 ± 0.01	18.07 ± 0.45	48.43 ± 3.13^*∗*^	24.14 ± 0.16^*∗*^	37.42 ± 0.03^*∗*^	50.51 ± 0.05^*∗*^	22.21 ± 0.40^*∗*^	33.32 ± 0.25^*∗*^	50.03 ± 0.38^*∗*^
LDL-C (g/dL)	66.07 ± 0.27	177.32 ± 2.45	67.17 ± 0.34^*∗*^	81.48 ± 0.33^*∗*^	75.30 ± 0.16^*∗*^	69.08 ± 0.24^*∗*^	84.60 ± 3.30^*∗*^	80.21 ± 0.31^*∗*^	73.35 ± 0.27^*∗*^
VLDL-C (mg/L)	11.79 ± 0.01	39.03 ± 1.08	11.85 ± 3.32^*∗*^	18.90 ± 0.01^*∗*^	12.78 ± 0.02^*∗*^	11.91 ± 0.02^*∗*^	22.25 ± 1.18^*∗*^	17.01 ± 0.28^*∗*^	12.63 ± 0.27^*∗*^

Values are expressed as mean ± SEM (*n* = 7); *p* < 0.05 was considered statistically significant. *∗* = statistical difference between treated groups and negative control. Aspartate aminotransferase, AST; alanine aminotransferase, ALT; alkaline phosphatase, ALP; total protein, TP; total bilirubin, TB; total cholesterol, TC; triacylglycerol, TAG; low density lipoprotein cholesterol, LDL-C; high density lipoprotein cholesterol, HDL-C; very low density lipoprotein cholesterol, VLDL-C.

## References

[1] Porth C. M. (1998). *Pathophysiology: Concepts of Altered Health States*.

[2] Cooke D. W., Plotnick L. (2008). Type 1 diabetes mellitus in pediatrics. *Pediatrics in Review*.

[3] Dorner M., Pinget M., Brogard M. J. (1977). Essential labile diabetes. *Munchener Medizinische Wochenschrift*.

[4] Kaminsky R., Ducray P., Jung M. (2008). A new class of anthelmintics effective against drug-resistant nematodes. *Nature*.

[5] Sofowara N. A. (2006). *Medicinal Plants and Traditional Medicine in Africa*.

[6] Areghore E. M., Makkar H. P. S., Becker K. (1997). Chemical composition and tannins in leaves of some browse plants from Delta (Central Nigeria) eaten by ruminants. *Proceedings of the Society of Nutrition Physiology*.

[7] Akah P. A., Okafor C. L. (1992). Blood sugar lowering effect of *Vernonia amygdalina* Del. in an experimental rabbit model. *Phytotherapy Research*.

[8] Alebachew M., Kinfu Y., Makonnen E., Bekuretsion Y., Urga K., Afework M. (2014). Toxicological evaluation of methanol leaves extract of *Vernonia bipontini* Vatke in blood, liver and kidney tissues of mice. *African Health Sciences*.

[9] Amole O. O., Izegbu M. C., Onakoya J. A. A., Dada M. O. (2006). Toxicity studies of the aqueous extract of *Vernonia amygdalina*. *Biomedical Research*.

[10] Asase A., Oppong-Mensah G. (2009). Traditional antimalarial phytotherapy remedies in herbal markets in southern Ghana. *Journal of Ethnopharmacology*.

[11] Ayoola G., Coker H., Adesegun S. (2008). Phytochemical screening and antioxidant activities of some selected medicinal plants used for malaria therapy in Southwestern Nigeria. *Tropical Journal of Pharmaceutical Research*.

[12] Akah P. A., Alemji J. A., Salawu O. A., Okoye T. C., Offiah N. V. (2009). Effects of *Vernonia amygdalina* on biochemical and hematological parameters in diabetic rats. *Asian Journal of Medical Sciences*.

[13] Kola O. (2007). Anti-inflammatory activity of ethanolic leaf extract from *Vernonia amygdalina* on the immune system of Swiss Albino rats dosed with *Clostridium sporogenes* (NC13532). *Research Journal of Medical Sciences*.

[14] Akah P. A., Njoku O., Nwanguma A., Akunyili D. (2008). Effects of aqueous leaf extract of *Vernonia amygdalina* on blood glucose and triglyceride levels of alloxan-induced diabetic rats (*Rattus rattus*). *Animal Research International*.

[15] Nwajo H. U. (2006). Efficacy of aqueous leaf extract of *Vernonia amygdalina* on plasma lipoproteins and oxidative status in diabetic rat model. *Nigerian Journal of Physiological Sciences*.

[16] Asante D., Ameyaw O. E., Effah-Yeboah E., Gyamenah A. P., Djabanor J. (2015). Hepatoprotective effect of ethanolic leaf extracts of *Abrus precatorius* in *Plasmodium berghei* infected imprinting control region (ICR) mice; a histopathological perspective. *International Journal of Biological and Pharmaceutical Research*.

[17] Boampong J. N., Karikari A. A., Ameyaw E. O. (2015). In vivo antiplasmodial and in vitro antioxidant properties of stem bark extracts of *Haematostaphis barteri*. *Asian Pacific Journal of Tropical Biomedicine*.

[18] Bancroft J. D., Gamble M. (2008). *Theory and Practice of Histological Techniques*.

[19] Grover J. K., Yadav S., Vats V. (2002). Medicinal plants of India with anti-diabetic potential. *Journal of Ethnopharmacology*.

[20] Zheng T., Shu G., Yang Z., Mo S., Zhao Y., Mei Z. (2012). Antidiabetic effect of total saponins from *Entada phaseoloides* (L.) Merr. in type 2 diabetic rats. *Journal of Ethnopharmacology*.

[21] Kunyanga C. N., Imungi J. K., Okoth M., Momanyi C., Biesalski H. K., Vadivel V. (2011). Antioxidant and antidiabetic properties of condensed tannins in acetonic extract of selected raw and processed indigenous food ingredients from Kenya. *Journal of Food Science*.

[22] Kim J.-S., Kwon C.-S., Son K. H. (2000). Inhibition of alpha-glucosidase and amylase by luteolin, a flavonoid. *Bioscience, Biotechnology and Biochemistry*.

[23] Mishra S. B., Raoch C. H. V., Ojha S. K., Vijayakumar M., Verma A. (2010). An analytical review of plants for anti diabetic activity with their phytoconstituent and mechanism of action. *International Journal Pharmaceutical Sciences and Research*.

[24] Wätjen W., Michels G., Steffan B. (2005). Low concentrations of flavonoids are protective in rat H4IIE cells whereas high concentrations cause DNA damage and apoptosis. *The Journal of Nutrition*.

[25] Szkudelski T. (2001). The mechanism of alloxan and streptozotocin action in *β* cells of the rat pancreas. *Physiological Research*.

[26] Kumar V., Ahmed D., Anwar F., Ali M., Mujeeb M. (2013). Enhanced glycemic control, pancreas protective, antioxidant and hepatoprotective effects by umbelliferon- *α*-D-glucopyranosyl-(2I→1II)-D glucopyranoside in streptozotocin induced diabetic rats. *SpringerPlus*.

[27] Khattab H. A. H., Al-Amoudi S. N., Al-Faleh A. A. (2013). Effect of ginger, curcumin and their mixture on blood glucose and lipids in diabetic rats. *Life Science Journal*.

[28] Ajagbonna O. P., Onifade K. I., Suleiman U. (1999). Haematological and biochemical changes in rats given extract of *Calotropis procera*. *Sokoto Journal of Veterinary Sciences*.

[29] Choudhari C., Deshmukh P. (2007). Acute and subchronic toxicity study of *Semecarpus anacardium* on hemoglobin percent and RBC count of male Albino rat. *Journal of Herbal Medicine and Toxicology*.

[30] Ojiako O. A., Nwanjo H. U. (2006). ‘Is *Vernonia amygdalina* hepatotoxic or hepatoprotective?’ response from biochemical and toxicity studies on rats. *African Journal of Biotechnology*.

[31] Branen A. L. (1975). Toxicology and biochemistry of butylated hydroxyanisole and butylated hydroxytoluene. *Journal of the American Oil Chemists' Society*.

[32] Verma P. R., Itankar P. R., Arora S. K. (2013). Evaluation of antidiabetic, antihyperlipidemic and pancreatic regeneration potential of aerial parts of *Clitoria ternatea*. *Brazilian Journal of Pharmacognosy*.

[33] Shepherd J. (2005). Does statin monotherapy address the multiple lipid abnormalities in type 2 diabetes?. *Atherosclerosis Supplements*.

[34] Shirwaikar A., Rajendran K., Barik R. (2006). Effect of aqueous bark extract of *Garuga pinnata* Roxb. in streptozotocin-nicotinamide induced type-II diabetes mellitus. *Journal of Ethnopharmacology*.

[35] Kumar V., Abbas A. K., Fausto A. N., Mitchell N. R. (2007). *Robbins Basic Pathology*.

[36] Mohan H. (2010). *Text Book of Pathology*.

[37] Hussein H. K., Abu-Zinadah O. A. (2010). Antioxidant effect of curcumin extracts in induced diabetic wister rats. *International Journal of Zoological Research*.

[38] Hashemnia M., Oryan A., Hamidi R. A., Mohammadalipour A. (2012). Blood glucose levels and pathology of organs in alloxan-induced diabetic rats treated with hydro- ethanol extracts of *Allium sativum* and *Capparis spinosa*. *African Journal of Pharmacy and Pharmacology*.

[39] Kumarappan C., Vijayakumar M., Thilagam E. (2011). Protective and curative effects of polyphenolic extracts from *Ichnocarpus frutescense* leaves on experimental hepatotoxicity by carbon tetrachloride and tamoxifen. *Annals of Hepathology*.

[40] Stevens A., Lowe J. S., Young B. (2002). *Wheater's Basic Histopathology: A Colour Atlas and Text*.

