# Corrigendum to “Antidiabetic Effect of Young and Old Ethanolic Leaf Extracts of *Vernonia amygdalina*: A Comparative Study”

**DOI:** 10.1155/2017/5618548

**Published:** 2017-12-04

**Authors:** Du-Bois Asante, Emmanuel Effah-Yeboah, Precious Barnes, Heckel Amoabeng Abban, Elvis Ofori Ameyaw, Johnson Nyarko Boampong, Eric Gyamerah Ofori, Joseph Budu Dadzie

**Affiliations:** ^1^Department of Biomedical Sciences, School of Allied Health Sciences, College of Health and Allied Sciences, University of Cape Coast, Cape Coast, Ghana; ^2^Department of Clinical Nutrition and Dietetics, School of Allied Health Sciences, College of Health and Allied Sciences, University of Cape Coast, Cape Coast, Ghana; ^3^Department of Biochemistry, School of Biological Sciences, College of Agriculture and Natural Sciences, University of Cape Coast, Cape Coast, Ghana

In the article titled “Antidiabetic Effect of Young and Old Ethanolic Leaf Extracts of *Vernonia amygdalina*: A Comparative Study” [1], there was an error in the unit of measurement included in the section “2.5. Preparation of Leaf Extract.” Thus, “The leaves were then grouped into young leaves (YL) and old leaves (OL) using the following criteria; length of YL ranges within 5.10–15.50 mm and width within 1.80–7.20 mm and their corresponding weights range within 0.09–1.20 g. Length of OL ranges within 17.70–30.10 mm and width within 7.20–12.90 mm, with corresponding weights ranging within 1.30–5.30 g,” should be corrected to “The leaves were then grouped into young leaves (YL) and old leaves (OL) using the following criteria: length of YL ranges within 5.10–15.50 cm and width within 1.80–7.20 cm and their corresponding weights range within 0.09–1.20 g. Length of OL ranges within 17.70–30.10 cm and width within 7.20–12.90 cm, with corresponding weights ranging within 1.30–5.30 g.”
